# Malathion increases apoptotic cell death by inducing lysosomal membrane permeabilization in N2a neuroblastoma cells: a model for neurodegeneration in Alzheimer’s disease

**DOI:** 10.1038/cddiscovery.2017.7

**Published:** 2017-04-24

**Authors:** Ramu Venkatesan, Yong Un Park, Eunhee Ji, Eui-Ju Yeo, Sun Yeou Kim

**Affiliations:** 1 College of Pharmacy, Gachon University, #191, Hambakmoero, Yeonsu-gu, Incheon 21936, Republic of Korea; 2 Vanta Bioscience, K3, 11(th) Cross Street, SIPCOT Industrial Complex, Gummidipundi 601 201, India; 3 Department of Biochemistry, College of Medicine, Gachon University, #191 Hambakmoero, Yeonsu-gu, Incheon 406-799, Republic of Korea; 4 Gachon Institute of Pharmaceutical Science, Gachon University, #191 Hambakmoero, Yeonsu-gu, Incheon 21936, Republic of Korea; 5 Gachon Medical Research Institute, Gil Medical Center, Inchon 21565, Republic of Korea

## Abstract

Malathion is an organophosphate with severe neurotoxic effects. Upon acute exposure, malathion initially enhances cholinergic activity by inhibition of acetylcholinesterase, which is its major pathological mechanism. Malathion also induces non-cholinergic neuronal cell death in neurodegenerative conditions; the associated molecular mechanism is not well-characterized. To investigate the molecular mechanism of malathion-induced cell death, N2a mouse neuroblastoma cells were exposed to malathion and cell death-related parameters were examined. Malathion reduced cell viability mainly by apoptosis through mitochondrial dysfunction in N2a cells, as judged by an increase in the level of the pro-apoptotic protein Bax and decrease in the levels of the anti-apoptotic proteins p-Akt and Bcl2, resulting in cytochrome *c* release and caspase-dependent DNA fragmentation and condensation. Malathion treatment also induced autophagy and lysosomal membrane permeabilization (LMP) in N2a cells. LMP caused a lessening of autophagic flux via inhibition of lysosomal fusion with the autophagosome. LMP-induced cathepsin B release and its proteolytic effect may intensify apoptotic insults. Moreover, malathion-exposed N2a cells showed a marked reduction in the levels of the neuronal marker proteins vascular endothelial growth factor and heart fatty acid binding protein 3, along with diminished neuritogenesis in N2a cells and nerve growth factor secretion in C6 glioma cells. Our data suggest that the non-cholinergic effect of malathion may be mediated by apoptotic cell death via LMP induction in N2a cells. Malathion-treated N2a cells can be utilized as an *in vitro* model system to screen natural and new chemical drug candidates for neurodegenerative diseases such as Alzheimer’s disease.

## Introduction

Alzheimer's disease (AD) is the most common neurodegenerative disorder. It is characterized by progressive memory loss and impaired cognitive ability.^1^ Predominant pathological markers of AD include amyloid plaque deposition, formation of neurofibrillary tangles, impaired synapses, microglial activation, cholinergic deficiency, oxidative stress, excitotoxicity, and mitochondrial dysfunction.^2^ Enormous numbers of research papers and reviews over the last century have demonstrated the remarkable etiology of AD and pointed out the age-related prevalence of neurodegeneration^3^ as well as many potentially related environmental hazards.^4^

Various environmental factors have been reported to induce neurodegenerative diseases like AD, Parkinson's disease, and amyotrophic lateral sclerosis.^5^ Primarily, pesticides, such as paraquat, maneb, rotenone, dieldrin, pyrethroids, and other organophosphates, have drawn attention.^6^ The WHO’s neurobehavioral test batteries for the evaluation of occupational field exposure recognize that organophosphate pesticide exposure increases the risk of cognitive dysfunction and vulnerability to neurodegeneration.^7,8^ Organophosphate pesticides are the most commonly used chemical agents for the control of pests in homes and in agriculture, such as insects and mites; contamination with such pests is associated with an increased risk of AD. A case–control study showing that the levels of pesticide traces from occupational exposure was higher in serum taken from AD patients than in serum from normal subjects suggested a direct link between environmental organophosphate pesticide exposure and AD.

Malathion (O, O-dimethyl thiophosphate of diethyl mercaptosuccinate) is an organophosphate that binds irreversibly to the two mammalian cholinesterases (ChE), acetylcholinesterase (AChE), and butyrylcholinesterase. Malathion is used worldwide in agricultural crops, such as lettuce, beans, broccoli, tomatoes, peaches, strawberries, and cherries, and also in residences for mosquito management.^9^ Malaoxon, a primary breakdown product of malathion, is estimated to be 60 times more toxic than malathion. The large numbers of commercial pursuits that involve extensive use of malathion have aroused scientific interest for investigation into neurodegenerative diseases. A case study showed that malathion was implicated in neuronal loss, and that its effect is mediated by irreversible inhibition of ChE. The effect of malathion exposure on cognitive dysfunction may also be mediated by apoptotic cell death through the promotion of pro-apoptotic proteins and mitochondrial protein release in adult mouse hippocampal neurons.^10^

Autophagy is an intracellular degradation process that recycles abnormal cytoplasmic proteins and organelles, and maintains homeostasis. Autophagy-derived intracellular materials are degraded in the acidic lumen of late endocytic compartments including lysosomes. Perturbation of autophagy and subsequent lysosomal degradation can lead to accumulation of abnormal protein aggregates, resulting in cellular dysfunction and disease states. Evidence from recent studies has revealed that autophagy is defective in most neurodegenerative diseases associated with accumulation of abnormal protein aggregates.^11^ Because defects in autophagy and lysosomal degradation can promote apoptotic neuronal cell death, which worsens neurodegeneration, pharmacological induction of autophagy may be a useful treatment strategy in neurodegenerative disorders.^12^

Lysosomes are filled with numerous hydrolases that degrade cellular macromolecules during the autophagic and endocytic processes. Lysosomal membrane permeabilization (LMP) can occur as a result of osmotic lysis or detergent activity of the compounds that accumulate in the lumen of lysosomes. Some endogenous compounds, including reactive oxygen species, lipid metabolites, such as sphingosine and phosphatidic acid, and some endogenous cell death effectors, such as Bax, can also cause LMP.^13^ Depending on the cellular context, the nature of the stimulus, and the extent of the leakage, LMP can initiate or amplify different types of lysosomal cell death including non-programmed (accidental) necrosis, lysosomal apoptosis, or cell death with apoptosis-like features.^14^ The lysosomal cell death pathway can be initiated by the release of cathepsins and other hydrolases from the lysosomal lumen to the cytosol.^13,15–17^ Cathepsins mediate proteolytic activation of pro-apoptotic proteins, such as Bax, and digestion/inactivation of anti-apoptotic Bcl2, resulting in mitochondrial membrane permeabilization, cytochrome *c* (cyto *c*) release, and caspase-dependent cell death.^14,18–20^

Because of the commercial availability and cytotoxicity of malathion, it is of interest to investigate the molecular mechanisms of neuronal cell death induced by malathion exposure and their roles in neurodegenerative diseases. The present study will focus on the roles of apoptosis, mitochondrial dysfunction, autophagy, and LMP in malathion-induced cytotoxicity in N2a cells. In addition, we will examine neurite outgrowth in malathion-treated N2a cells and nerve growth factor (NGF) secretion from C6 glioma cells treated with N2a-conditioned media in order to establish an *in vitro* AD model.

## Results

### Malathion induces cytotoxicity of N2a cells

**Figure 1:**
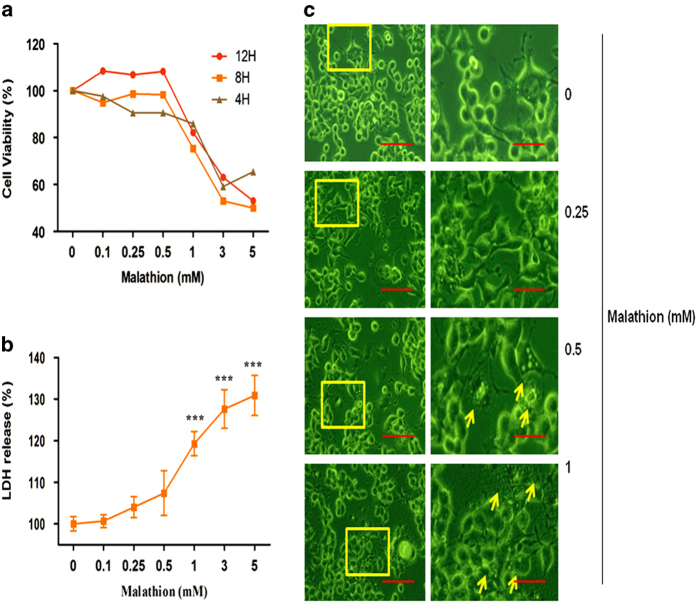
Malathion-induced neurotoxicity in N2a cells. (**a**) N2a cells were treated with vehicle (0) or various concentrations (0.1–5 mM) of malathion for 4–12 h and cell viability was measured with an MTT assay. (**b**) After 8 h of malathion treatment, the morphology of N2a cells was observed and photographed using a phase-contrast microscope (200 *μ*m); magnified images (50 *μ*m) are also shown. (**c**) Malathion (0.1–5 mM) for 8-h-induced cytotoxicity in N2a cells was measured by an LDH release assay. All data in **c** are shown as mean±S.D. ***P<0.001 compared with vehicle-treated control cells.

To determine the effect of malathion on neuronal cell viability, N2a cells were treated with vehicle (dimethyl sulfoxide: DMSO) or varying concentrations (0.1–5 mM) of malathion for the indicated times (4, 8, and 12 h) and cell viability was measured with an MTT assay. As shown in Figure 1a, malathion treatment reduces N2a cell viability in a dose- and time-dependent manner. Because 1 mM malathion reduced cell viability to 70–80% at different time points and 8 h treatment was the most effective in terms of reducing cell viability, the following experiments were performed with 8 h malathion treatment.

Morphological changes after malathion treatment (0.25–1 mM) were also observed under a phase-contrast microscope (Figure 1b). Observations of cell morphology revealed that malathion induced cytotoxicity in N2a cells. To confirm the cytotoxic effect of malathion, N2a cells were treated with various concentrations of malathion (0.1–5 mM) for 8 h, and lactate dehydrogenase (LDH) activity in the culture supernatant was measured using the LDH assay kit. Cytotoxicity data are expressed as the percentage of LDH activity in the experimental media as compared with the control. Our data showed that malathion treatment increased LDH release in a dose-dependent manner (Figure 1c). These results indicate that exposure to malathion in the environment may induce neurotoxicity.

### Malathion induces apoptotic cell death via mitochondrial dysfunction in N2a cells

**Figure 2:**
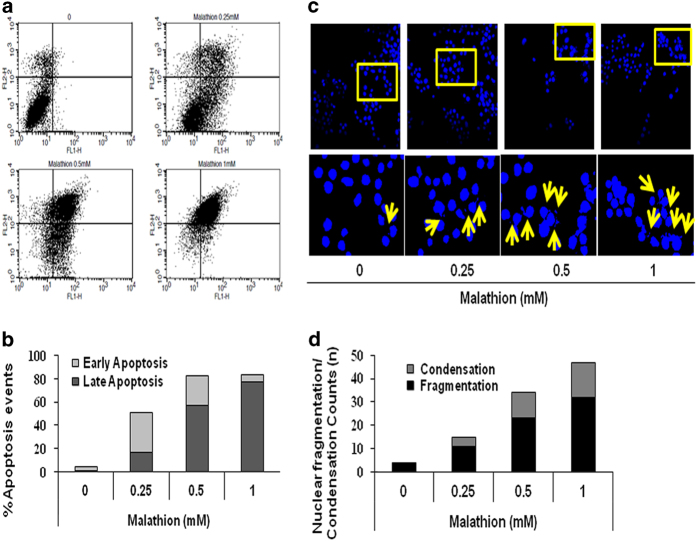
Malathion induces apoptotic cell death in N2a cells. (**a**) N2a cells were treated with vehicle (0) or malathion (0.25–1 mM) and examined for apoptosis by flow cytometry after being stained with the annexin V-FITC apoptosis detection kit. (**b**) The graphical representation of malathion-induced apoptosis (%) shows that many cells shifted from the left lower to the right lower quadrant (early apoptosis) and to the right upper quadrant (late apoptosis). (**c**) N2a cells were treated with malathion (0.25–1 mM) for 8 h and nuclear morphological changes, such as nuclear disintegration and fragmentation, were examined by staining of nuclei with Hoechst 33258. Condensed and fragmented nuclear materials in N2a cells were examined under a confocal microscope (×40). The red arrows indicate condensed and fragmented nuclei. (**d**) The number of condensed and fragmented nuclei was counted after 8 h exposure to malathion, and the number per 200 N2a cells was plotted.

Apoptosis plays a central role in neuronal cell death. To examine whether malathion induces apoptotic cell death, N2a cells were treated with malathion (0.25, 0.5, or 1 mM) for 8 h and cells were stained using the annexin V-fluorescein isothiocyanate (FITC) apoptosis detection kit for flow cytometric analysis. The flow cytometric data revealed that malathion increased the percentage of cells in both early (lower right quadrant) and late apoptosis (upper right quadrant). The percentage of apoptosis events in the two stages differed depending on the dose of malathion. Lower concentrations of malathion (0.25 mM) increased the percentage of early apoptosis events, whereas higher concentrations of malathion (1 mM) increased the percentage of late apoptosis events (Figure 2b). Late apoptosis might be responsible for LDH leakage at higher concentrations of malathion.

Malathion-induced apoptotic cell death was confirmed by nuclear DNA fragmentation/chromatin condensation. N2a cells were stained with the blue fluorescent DNA binding dye Hoechst 33258 and nuclear morphology was examined. As shown in Figure 2c, nuclear morphological changes subsequent to malathion treatment (0.25, 0.5, or 1 mM) were readily apparent. The number of cells with DNA fragmentation and chromatin condensation (hallmarks of disintegrating nuclei) increased in a dose-dependent manner (Figure 2d).

**Figure 3:**
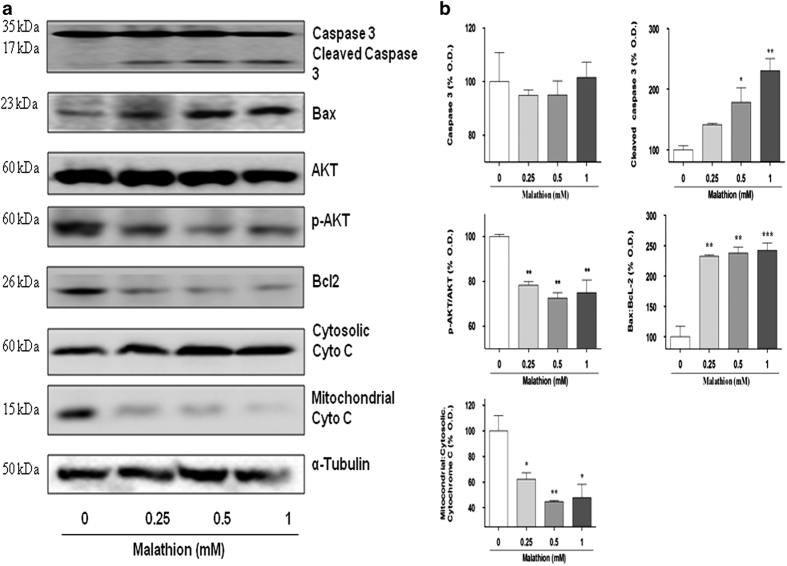
Malathion alters the levels of apoptosis-related proteins in N2a cells. N2a cells were treated with vehicle (0) or malathion (0.25–1 mM) for 8 h and the cell lysate was prepared. (**a**) Levels of intact caspase 3, cleaved caspase 3, the pro-apoptosis protein Bax, the anti-apoptotic proteins p-Akt/Akt and Bcl2, and cytosolic/mitochondrial cyto *c* in malathion-treated cells were examined by Western blot analysis using the corresponding antibodies. *α*-Tubulin was used as a loading control. (**b**) The bar graph represents densitometric analysis of the given protein levels after malathion treatment. All data are shown as the mean±S.D. of three independent experiments. *P<0.05 and **P<0.01 compared with vehicle-treated control cells.

Caspase 3 is one of the executive caspases that cleave DNA during apoptotic cell death. To confirm that apoptotic cell death occurred in malathion-treated N2a cells, the levels of intact and cleaved caspases were analyzed by Western blot. As expected, the active form of caspase 3 (cleaved caspase 3) was increased (141.3, 178.4, 230.4%) by treatment with increasing concentrations of malathion (Figure 3). In addition, malathion (0.25, 0.5, 1 mM) increased the level of the pro-apoptotic protein Bax (166.5, 167.0, 170.3%) and decreased the levels of the anti-apoptotic proteins p-Akt (71.7, 65.2, 69.2%) and Bcl2 (70.4, 68.9, 69.0%; Figure 3). Because these proteins are implicated in mitochondrial dysfunction, cyto *c* release from the mitochondria to the cytosol was further examined. As shown in Figure 3, the level of cytosolic cyto *c* (103.1, 143.9, 126.9%) increased but that of mitochondrial cyto *c* (64.1, 62.8, 55.5%) decreased in malathion-treated cells. Taken together, the present data suggest that malathion induces cytotoxicity via mitochondrial dysfunction-associated apoptotic cell death.

### Malathion induces autophagy in N2a cells

**Figure 4:**
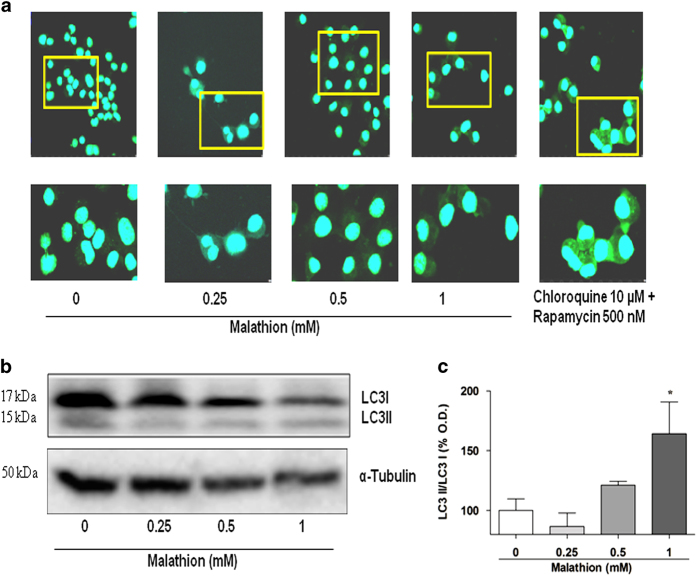
Autophagy is induced by malathion in N2a cells. (**a**) N2a cells were treated with vehicle (0) or varying concentrations of malathion (0.25–1 mM) for 8 h and assessed for induction of autophagy by staining with a Cyto-ID autophagy detection kit. N2a cells were also treated with a mixture of chloroquine (10 *μ*M) and rapamycin (500 nM) as positive controls. Green fluorescence was analysed by confocal microscopy. (**b**) Malathion-treated N2a cells were examined for the expression of the autophagy-associated proteins LC3I and II by Western blot analysis. (**c**) The bar graph represents the densitometric analysis of LC3 protein expression after malathion treatment. All data are shown as mean±S.D. *P<0.05 compared with vehicle-treated control cells.

Because many cytotoxic insults can cause both apoptosis and autophagy under certain conditions, we investigated the effect of malathion on autophagy in N2a cells. To assess autophagy, autophagic vacuoles were detected by staining with a novel dye, Cyto-ID, and observed under a confocal microscope (Figure 4a). The intensity of green fluorescence in Figure 4a revealed that malathion induced autophagy in N2a cells in a dose-dependent manner. However, the fluorescence intensity in 1 mM malathion-treated cells was weaker than that in the positive controls treated with chloroquine (10 *μ*M) and rapamycin (500 nM).

Autophagic induction by malathion was verified by a change in the levels of the autophagic marker protein LC3B. The level of LC3B was examined by Western blot analysis with LC3B antibodies. Figure 4b shows that the levels of both LC3B I and II proteins decreased with increasing concentrations of malathion (0.25–1 mM). The changes in LC3B proteins may represent an autophagic flux into the lysosome in malathion-treated N2a cells. The ratio of LC3B II/LC3B I, an indicator of the autophagic flux rate, was calculated and plotted. The ratio decreased with 0.25 mM malathion treatment, indicating rapid autophagic flux and degradation of autophagosomes (Figure 4c). However, the ratio increased with malathion treatment at 1 mM, which indicates a retardation of autophagic flux. The data suggest that malathion affects autophagy differentially depending on treatment dose.

### Malathion induces LMP, which counteracts autophagy to accelerate apoptotic cell death in N2a cells

**Figure 5:**
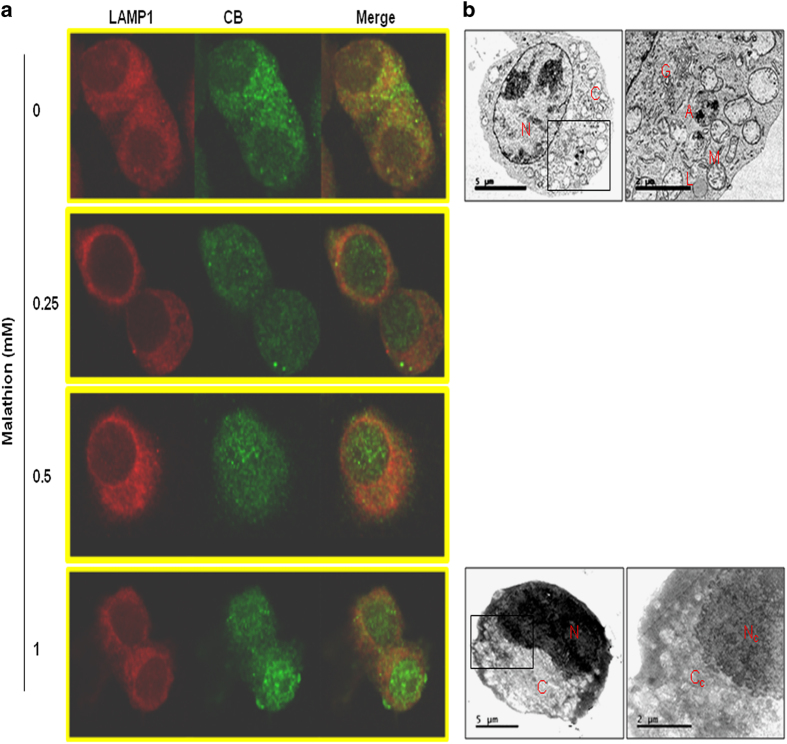
LMP is induced by malathion. (**a**) N2a cells were treated with vehicle or varying concentrations of malathion (0.25–1 mM) for 8 h and analysed for LMP using immunofluorescence staining. Cells were fixed and treated with anti-LAMP1 and anti-CB antibodies and then treated with the corresponding secondary antibodies in order to detect LAMP1 (red) and CB (green). The images were merged to detect the release of CB from the lysosome to the cytosol or nucleus. (**b**) After fixation with 2.5% glutaraldehyde solution, cells were prepared into ultra-thin sections and subjected to TEM imaging to visualize cells and cellular organelles. The vehicle-treated controls exhibit clear and intact cells and cellular organelles; the lysosome can be seen in the cytosol and there is no damage to the nucleus or nuclear membrane. Malathion (1 mM)-treated cells show total collapse of cellular organelles and the extrusion of nuclear materials into the cytosol. A, autophagosome; C, cytosol; Cc, collapsed cytosol; G, Golgi body; L, lysosome; M, mitochondria; N, nucleus; Nc, collapsed nucleus.

LMP is one of the factors that control apoptotic cell death. In this study, we examined whether malathion affects LMP in N2a cells. LMP was assessed by immunofluorescence staining using primary antibodies against the lysosomal membrane protein LAMP1 and the lysosomal hydrolytic enzyme marker cathepsin B (CB), and secondary antibodies with fluorescent tags. CB (green) co-localized to the lysosomes along with LAMP1 (red) in vehicle-treated control cells, as judged by increased yellowish fluorescence in the merged photos (Figure 5a). However, malathion treatment markedly increased the intensity of green fluorescence (CB) in the cytosol and nucleus. This result indicates that malathion induces lysosomal destabilization and causes CB release.

To confirm the malathion-induced changes in LMP in N2a cells, treated cells were fixed with glutaraldehyde and ultra-thin sections were examined using transmission electron microscopy (TEM) imaging (Figure 5b). The results of TEM revealed that malathion (1 mM) treatment caused total destruction of the cell and cellular organelles. The images (2 *μ*m) illustrate that loss of nuclear membrane integrity leads to the flow of nuclear material from the nucleus to the cytosol. In addition, the organelles in the vehicle-treated control cells were observed to have intact and clearly delineated margins; mitochondria, lysosomes, autophagosomes, and golgi bodies can be seen. Malathion treatment caused complete collapse of cellular organelles, clearly illustrating that damage to the LMP and associated spillage of lysosomal contents effectively destroyed the cell.

### Malathion hampers neurite outgrowth and the expression of neuronal proteins in N2a cells, and NGF secretion in C6 glioma cells

**Figure 6:**
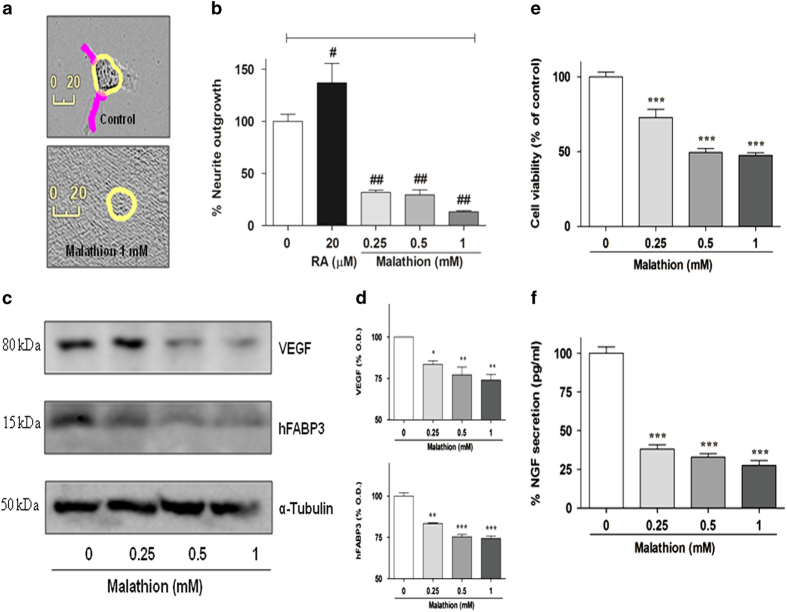
Effect of malathion on neurite outgrowth, neuronal protein expression in N2a cells, and NGF secretion in C6 glioma cells. (**a**) The effect of malathion on neurite outgrowth was examined after 8 h treatment of N2a cells with vehicle (upper) or 1 mM malathion (lower). Malathion-treated N2a cells were photographed and neurites were drawn. (**b**) Neurite length in malathion-treated cells was measured using IncuCyte and plotted, along with neurites of vehicle-treated and retinoic acid (RA; positive control)-treated cells. All data are shown as mean±S.D. of triplicate experiments. *P<0.05 and **P<0.01 compared with vehicle-treated control cells. (**c**) N2a cells were exposed to malathion (0.25–1 mM) for 8 h and examined for the expression of two neuronal proteins, VEGF and hFABP3, by Western blot. (**d**) Bar graph showing the results of densitometric analysis of VEGF and hFABP3 protein expression, which were altered by malathion treatment in a concentration-dependent manner. *P<0.05, **P<0.01, and ***P<0.001 compared with vehicle-treated control cells. (**e**) C6 glioma cells were conditioned with media from N2a cells that were treated with malathion (0.25–1 mM) for 8 h. Cell viability (%) was assessed for 24 h in C6 glioma cells. (**f**) At 24 h after exposure to conditioned media, the media was collected and examined for NGF secretion using ELISA. ***P<0.001 compared with vehicle-treated control cells.

Because malathion caused neuronal cytotoxicity, our attention turned to its effect on neuronal function. We first examined neurite outgrowth in order to assess neuronal connectivity. Malathion treatment reduced neurite length in N2a cells when compared with vehicle-treated controls and retinoic acid-treated positive controls in a dose-dependent manner (Figures 6a and b). The results suggest that malathion hampers neuritogenesis and thus neuronal connectivity.

We next examined whether malathion affects the expression of certain proteins that are important for neuronal function. Vascular endothelial growth factor (VEGF), a well-known molecule involved in vascular integrity, has been shown to be essential in many neurons for normal function, growth, and integrity.^21^ Because a decreased VEGF level was identified as a marker of neurodegeneration in diabetic retinopathy, we examined VEGF levels in malathion-treated cells. In addition, we also examined the level of fatty acid binding/transporting protein hFABP3, because transportation of fatty acids to the mitochondria is essential in fulfilling the energy requirements of the brain. Deficiencies in fatty acid transporter proteins lead to unbalanced energy production and cause neurodegeneration and other aging-related impairments.^22^ Our results clearly showed that the expression of VEGF and hFABP3 was decreased by treatment with increasing concentrations of malathion (Figures 6c and d).

It was previously reported that neuritogenesis depends on neurotrophic growth factors secreted by astrocytes. So, we investigated the level of soluble NGF secreted by C6 glioma cells. N2a cells were treated with malathion and then the culture media was collected and used to culture C6 glioma cells for 24 h, at which point cell viability and soluble NGF secretion were measured. Malathion-treated N2a media reduced the viability (%) of C6 glioma cells in a dose-dependent manner (Figure 6e). Malathion-treated media also reduced NGF secretion from C6 cells when compared with vehicle-treated control media (Figure 6f). Malathion-induced reduction of neuritogenesis, neuronal protein expression, and NGF secretion indicate that malathion may play a role in the induction of neurodegenerative diseases such as AD.

## Discussion

Environmental toxins, such as pesticides and metals, contribute to pollution in the ecosystem.^23^ According to the United States Environmental Protection Agency (US-EPA), roughly 1.2 billion pounds of pesticides are used in the USA every year for residential, agricultural, industrial, and government purposes. Pesticides are known to cause severe damage to the environment and ground water systems. Malathion, an organophosphate pesticide, is widely used for many commercial purposes on an everyday basis. The Agency for Toxic Substances and Disease Registry (ATSDR) and the US-EPA reported on the systemic effect of malathion on AChE, which exerts its effect on nerve ends of central, peripheral, somatic, and autonomic nervous systems.^24^ Malathion facilitates neurotoxicity by direct interaction with and inhibition of ChE, thereby mediating organ-specific toxicity in the liver, kidneys, heart, and lungs.^25^ Consequently, malathion causes neuromuscular junction-mediated fasciculation, cramps, diminished tendon reflexes, muscle weakness, ataxia, and paralysis.

Although the anti-cholinergic effect of malathion has been reported to induce neurotoxicity in neurodegenerative disease states, a non-cholinergic mode of cell injury, neuronal cell death, is also involved in malathion-induced neurotoxicity. Acute exposure of N2a mouse neuroblastoma cells to malathion reduced cell viability by inducing cytotoxicity (Figure 1) and apoptotic cell death (Figure 2). Malathion-induced apoptosis is mediated by increased levels of pro-apoptotic proteins, such as Bax and cleaved caspase 3, and by decreased levels of anti-apoptotic proteins such as p-Akt and Bcl2 (Figure 3). Malathion-induced release of cyto *c* from the mitochondria to the cytosol indicates the occurrence of mitochondrial membrane permeabilization and mitochondrial dysfunction.

Our data are in agreement with that of a previous report on chlorpyrifos-induced neurotoxicity. Chlorpyrifos is another organophosphate that induces apoptotic cell death by increasing the level of Bax and decreasing the level of Bcl2, followed by cyto *c* release and caspase 3 activation, in SH-SY5Y cells.^12^ Activation of NF-*κ*B through the p53 signaling pathway contributes to chlorpyrifos-induced changes in apoptosis-associated proteins in human neural precursor cells.^26^ Chronic exposure to the herbicide atrazine also induces mitochondrial dysfunction via a reduction in oxidative phosphorylation in the liver and skeletal muscles in rats.^27^ Mitochondrial dysfunction-induced apoptotic cell death may be a generalized mechanism for pesticide-induced cytotoxicity.

The autophagy machinery is known to promote cell survival by formation of autophagosomes containing worn-out proteins and organelles targeted for lysosomal degradation. Autophagic activity can be monitored by Atg8/LC3 activity in mammalian cells.^28^ Lysosomal fusion with autophagosomes and associated catabolism leads to a rapid downfall in LC3B protein levels.^29^ As malathion treatment increased the activity of the autophagy marker Cyto-ID (Figure 4a) and reduced the level of LC3B II (Figure 4b), we suggest that malathion induces autophagy, thereby consuming LC3B II proteins, in N2a cells. In addition, defective autophagic activity can cause a reduction of LC3B I protein expression and maintenance of LC3B II proteins.^30^ In the present study, the ratio of LC3B II/LC3B I was shown to be reduced by a low concentration (0.25 mM) but increased by a high concentration (1 mM) of malathion (Figure 4c). These results suggest that malathion-induced autophagy is modulated differentially depending on treatment dose. Autophagic induction might be protective against apoptotic cell death at lower concentrations of malathion but not functional at higher concentrations of malathion in N2a cells.

To clarify the role of autophagy in malathion-induced cell death, lysosomal function was further assessed by measuring LMP. For the first time, the present study showed that malathion (0.25–1 mM) induced LMP, resulting in the release of the lysosomal lumen proteolytic enzyme CB in N2a cells and also widespread destruction of cellular organelles (Figure 5). Cathepsins have been shown to mediate the activation of pro-apoptotic proteins, such as Bid and Bax, and the inactivation of anti-apoptotic Bcl2 and Bcl-XL, resulting in mitochondrial outer membrane permeabilization, cyto *c* release, and apoptosome-dependent caspase activation.^13,14,18–20^ Although cathepsins are known to induce the proteolytic activation of caspases, massive LMP often results in cell death without caspase activation.^13,15–17^ LMP-induced CB release may cause a defect in lysosomal fusion with the autophagosome, resulting in mitochondrial dysfunction and cyto *c* release (Figure 3). The released cyto *c* may then trigger the intrinsic apoptosis pathway and cause activation of caspase 3.

Similarly, hydroxychloroquine treatment was reported to increase LMP induction and CB release, which was responsible for apoptotic cell death in pcDNA3.1, human Bcl2 vector-transfected HeLa, and BJAB cells.^31^ In addition, CB released from the lysosome was shown to initiate DNA damage owing to acidic cytosolic conditions.^13,20^ Malathion also induced DNA fragmentation and condensation in Hoechst 33258-stained N2a cells (Figures 2c and d), presumably via LMP-induced CB release. As described previously,^32^ LMP-induced mitochondrial dysfunction and DNA damage seem to enhance cell death by making cells overcome the protective autophagic conditions. Although it is difficult to prove that LMP is a main trigger of mitochondrial dysfunction and apoptotic cell death in malathion-treated cells, further studies on LMP may allow for the discovery of therapeutic drugs for neurodegenerative diseases.

The relationship between autophagy and apoptotic cell death is not entirely clear. It was previously reported that pre-treatment with the autophagy inducer rapamycin protected chlorpyrifos-induced apoptotic cell death, whereas pre-treatment with the autophagy inhibitor, 3-methyladenine, increased chlorpyrifos cytotoxicity in SH-SY5Y cells, suggesting an anti-apoptotic function of autophagy.^12^ In addition, induction of LC3 protein by potential autophagy enhancers protected SH-SY5Y cells from apoptosis caused by the organofluoride insecticide fipronil.^33^ However, following neuronal autophagy induction by rapamycin, the rate of apoptosis increased in another AD cell model, SH-SY5Y cells overexpressing the 695-amino-acid Swedish mutant of A*β* precursor protein (APP695swe), suggesting a pro-apoptotic function for autophagy.^34^ In the latter APP695swe-transfected AD cell model, memantine (5 *μ*M) increased neuronal cell survival by inhibiting neuronal autophagy and apoptosis via both mammalian target of rapamycin-dependent and -independent autophagic signaling pathways.^34^ In rotenone-treated SH-SY5Y cells, rotenone attenuated autophagic LC3 and beclin 1 levels, which were recovered by 1,25-dyhydroxyvitamin D_3 _(calcitriol).^35^ Rotenone-induced neurodegeneration involves initiation of autophagic flux before cells undergo apoptotic cell death.^35,36^ Like rotenone, malathion also induced autophagic flux (Figure 5) as well as apoptotic cell death (Figure 2) in N2a cells, suggesting that malathion might be useful in generating a neurodegenerative disease model. Malathion-exposed N2a cells can be utilized to search for potential therapeutic compounds for AD treatment, as shown by memantine and calcitriol, which have anti-autophagic and anti-apoptotic functions.

VEGF is an important growth factor in many retinal cells, including different types of neurons. Sustained intraocular VEGF neutralization induced retinal neurodegeneration and vascular damage in the diabetic eye^21^ via disturbance of the blood–retina barrier and vascular leakage of proteins such as albumin.^37,38^ A reduction in hFABP3 can cause neurodegeneration and other age-related impairments via unbalanced energy production.^22^ A possible role of malathion in neurodegeneration was investigated in the present study. We showed that malathion caused a host of neurodegeneration-related events such as blockage of neurite outgrowth, reduced expression of the neuronal proteins VEGF and hFABP3 in N2a cells, and reduced secretion of NGF in C6 glioma cells (Figure 6). Similarly, chlorpyrifos and parathion treatment in young rats was reported to decrease neurotrophic factors, such as NGF and brain-derived neurotrophic factor, in the hippocampus and cerebral cortex.^39^ NGF-mediated neuroprotection via the TrkA receptor enhances neurite outgrowth in human neuroblastoma cells, which is blocked by organophosphate drugs.^40^ Unfavorable environmental conditions surrounding neuronal cells may promote neuronal cell death.

In summary, malathion treatment causes apoptosis via mitochondrial dysfunction in N2a neuroblastoma cells. Malathion also upregulates the autophagy machinery, which would allow cells to withstand or recover from the toxic situation, but malathion-induced lysosomal destabilization causes a release of proteolytic CB, which worsens the apoptotic condition. Malathion also reduces the levels of VEGF and hFABP3, and thus causes neurodegeneration.

## Materials and Methods

### Reagents and antibodies

Malathion, protease inhibitor cocktail, and phosphatase inhibitor cocktail were purchased from Sigma-Aldrich (St. Louis, MO, USA). The Annexin V-FITC apoptosis detection kit was obtained from Santa Cruz Biotechnology (Dallas, TX, USA), Dulbecco’s modified Eagle medium (DMEM) was purchased from Thermo Scientific (Seoul, Korea), and fetal bovine serum (FBS) was obtained from Atlas Biologicals (Fort Collins, CO, USA). The Bio-Rad DC protein assay kit and LDH assay kit were obtained from Bio-Rad Laboratories (Seoul, Korea) and Roche Diagnostics (GMBH, Mannheim, Germany), respectively. The Cyto-ID autophagy detection kit was purchased from Enzo Life Science (Farmingdale, NY, USA). Primary antibodies against caspase 3, Akt, and p-Akt were purchased from Cell Signaling (Danvers, MA, USA). Antibodies against VEGF, Bax, Bcl2, CB, and LAMP1 were obtained from Santa Cruz Biotechnology. Anti-cyto *c*, hFABP3, LC3B, and *α*-tubulin antibodies were obtained from BD Bioscience (San Jose, CA, USA), Proteintech (Chicago, IL, USA), Abcam (Cambridge, MA, USA), and Sigma-Aldrich, respectively. FITC-conjugated (Alexa Fluor 488 conjugate) and Rhodamine-conjugated (Alexa Fluor 594 conjugate) secondary antibodies were obtained from Thermo Fisher Scientific (Rockford, IL, USA). All antibodies were diluted and used as recommended by the manufacturers.

### Cell culture and treatment

N2a mouse neuroblastoma cells were obtained from the Korean Cell Line Bank (Seoul, Korea). N2a cells were cultured in high-glucose DMEM supplemented with 10% FBS and 1% antibiotics (penicillin and streptomycin). Cultures were maintained in a humidified incubator with 5% CO_2_ at 37°C. Cells were seeded in 24- or 96-well plates at a density of 2 or 5×10^4^ cells per ml and incubated overnight before various analyses. Malathion treatment was carried out using 2% FBS in DMEM at various concentrations and an 8 h exposure time. Because malathion was dissolved in DMSO, control cells were treated with the vehicle DMSO.

### Cell viability assay

N2a cell viability was determined by the MTT (3-(4,5-dimethylthiazol-2-yl)-2,5-diphenyl tetrazolium bromide) assay, which is based on the colorimetric change of MTT to MTT formazan, a purple product, by mitochondrial enzymes. For that experiment, N2a cells were seeded in a 96-well plate at a density of 5×10^4^ cells per well and incubated overnight. Cells were then treated with various concentrations of malathion (0.1, 0.25, 0.5, 1, 3, and 5 mM) and incubated for the indicated times (4–12 h). Cells were washed once with PBS and then exposed to 0.5 mg/ml MTT solution for 1 h. The formazan product was solubilized using DMSO and the absorbance at 570 nm was read in an ELISA reader (Molecular Devices, Sunnyvale, CA, USA). Data are expressed as the percentage viable cells in the experimental condition compared to the vehicle-treated control.

### LDH release assay

N2a cells were seeded on a 96-well plate and treated with malathion as described previously. The LDH leakage to the culture medium, an indicator of cytotoxicity leading to cell death, was assessed using an LDH assay kit. After the treatment, the culture supernatant was collected and cells were removed by centrifugation at 10 000×*g*. The cell-free supernatant was incubated with the substrate mixture from the kit. LDH activity was determined in a coupled enzymatic reaction; during this reaction, the iodotetrazolium dye was reduced to formazan. The experiment was conducted as described in the user manual. The reaction was performed in the dark for 30 min and stopped using 1 N HCL solution, and the absorbance was measured at 490 nm. Cytotoxicity data are expressed as the percentage of LDH release in the culture supernatant over control.

### Evaluation of neurite outgrowth

Neurite outgrowth in N2a cells was assessed following malathion treatment. N2a cells were seeded at a cell density of 1×10^4^ per well in 6-well plates and treated with malathion (0.25, 0.5, or 1 mM) for 8 h. Automated images were captured using an Essen IncuCyte ZOOM v2013B Rev1 (Essence Bioscience Inc., Ann Arbor, MI, USA). Retinoic acid was used as a positive control to induce neurite outgrowth. The collected images were analyzed to measure neurite length, and the percent neurite outgrowth was calculated and plotted.

### Flow cytometry for annexin V-FITC apoptosis detection

Apoptotic cell death was detected by flow cytometry using the annexin V-FITC apoptosis detection kit. Neuro-2a cells were seeded at 5×10^4^ cells per ml in 100 mm dishes and treated with malathion (0–1 mM) for 8 h. Cells were detached from the dishes by trypsinization and collected by centrifugation at 1000×*g* for 5 min. After resuspension in annexin V-FITC binding buffer, cells were incubated with 1 *μ*g/ml annexin V-FITC and 10 *μ*g/ml PI at room temperature in the dark for 15 min. Samples were analyzed using a FACS Calibur flow cytometer (Becton Dickinson Biosciences, San Jose, CA, USA).

### Western blotting analysis

After the treatment period, cells were washed three times with PBS, then harvested using a cell scraper and a low speed (1000×*g*) centrifuge. The pellet was lysed using RIPA lysis buffer (50 mM Tris, pH 7.5, 150 mM NaCl, 0.1% sodium dodecyl sulfate, 0.5% deoxycholate, and 1% NP40) containing 1 mM phenylmethylsulfonyl fluoride, protease inhibitor cocktail, and phosphatase inhibitor cocktail for 30 min on ice. The cells in the lysis buffer were sonicated on ice and subjected to centrifugation at 12 000 rpm for 20 min at 4°C. The supernatant was collected and protein concentration was assessed using the Bradford reagent Bio-Rad DC kit. Samples with 25 *μ*g protein were separated by SDS-PAGE and blotted on nitrocellulose membranes. The membranes were blocked with 5% skim milk in TBST for 1 h, followed by overnight incubation with primary antibodies in 5% skim milk/TBST at 4°C in a shaker. The membranes were washed with TBST buffer three times and then incubated with secondary antibody (1: 5000) in 5% skim milk/TBST for 1 h at room temperature. Protein bands were visualized using an ECL detection system and band intensity was measured by Image Lab software (BioRadiations Life Science Research, Hercules, CA, USA). Protein levels were normalized to *α*-tubulin.

### Nuclear staining analysis by fluorescence microscopy

DNA damage was measured by nuclear staining with Hoechst 33258, which binds to AT-rich DNA. N2a cells (2×10^4^) were seeded onto glass coverslips in 24-well plates and incubated overnight. The cells were treated with various concentrations of malathion (0, 0.25, 0.5, or 1 mM) for 8 h. Cells were washed with PBS and fixed with 4% paraformaldehyde (PFA) for 20 min at room temperature. Cells were washed again with PBS and then stained with Hoechst solution in the dark at room temperature for 30 min. The stained nuclei were observed in a confocal laser scanning microscope (Nikon A1+, Tokyo, Japan) and photographed. The number of condensed/fragmented nuclei in 200 cells was counted and plotted.

### Quantification of NGF secretion by ELISA

To examine the effect of malathion on NGF secretion in C6 glioma cells, N2a cells (5×10^4^) were first treated with malathion (0–1 mM) for 8 h. The N2a cell media was collected and transferred to 24-well plates seeded with C6 cells (1×10^4^). After 24 h treatment with N2a media, the C6 culture media was collected and analyzed for NGF secretion.^41^ NGF quantification was performed as described in the protocol supplied by the manufacturer of the *β*-NGF ELISA kit (R&D system, Minneapolis, MN, USA). NGF levels are expressed in pg/ml.

### Autophagy detection assay

N2a cells (2×10^4^) were seeded onto glass coverslips in 24-well plates and incubated overnight. The cells were treated with various concentrations of malathion (0, 0.25, 0.5, or 1 mM) for 8 h. Autophagic induction was detected using the Cyto-ID autophagy detection kit. Along with malathion, cells were also treated with a mixture of 500 nM rapamycin and 10* μ*M chloroquine, included in the kit, as positive controls. Experiments were carried out according to the manufacturer’s instructions. Fluorescent images were captured with a confocal laser scanning microscope (Nikon A1+) with excitation and emission wavelengths of 488 and 530 nm, respectively. The induction of autophagy was assessed by the intensity of green fluorescence in the cytosol.

### Detection of LMP by immunocytochemistry

N2a cells were seeded onto glass coverslips in 6-well plates and incubated overnight. Cells were treated with malathion as described previously. Cells were washed once with PBS (pH 7.4) and fixed with 4% PFA for 20 min at room temperature. Cells were washed again with PBS and blocked in 1% horse/goat serum and 3% triton X-100 in PBS for 1 h at room temperature. Cells were then inspected for the location of LAMP1 and CB using anti-LAMP1 (mAb) and anti-CB (pAb), respectively. Cells were incubated with the primary antibodies overnight in a shaker at 4°C and washed twice with PBS. Next, cells were incubated with FITC-conjugated (Alexa Fluor 488 conjugate) and Rhodamine-conjugated (Alexa Fluor 594 conjugate) secondary antibodies at 1 : 200 dilution at room temperature in the dark for 2 h. Finally, cells were washed three times with PBS and mounted on slides. Images of the stained cells were captured with a confocal laser scanning microscope (Nikon A1+).

### TEM analysis of lysosomal morphology

To confirm induction of LMP by malathion, lysosomal morphological analysis was performed based on the method reported by Kang *et al.*^41^ To examine lysosomal integrity, the N2a cells were treated as described previously. At the end of the treatment period, cells were immersed in fixative (PBS-buffered 2.5% glutaraldehyde) and incubated overnight at 4°C. In addition, specimens were dehydrated by drenching in buffered 2% osmium tetroxide for 2 h at 4°C then embedded in resin followed by 50–60 nm thin sections were taken and stained with uranyl acetate and lead citrate. Lysosomal morphology was examined using Jeol 1400 transmission electron microscopy (Jeol Ltd, Tokyo, Japan). Sample preparation was done and the TEM instrument facility was provided by the Korean Basic Science Institute (KBSI, Chungbuk Ochang Center, Ochang, South Korea).

### Statistical analysis

Data are presented as the mean±S.D. of at least three independent experiments. Statistical comparisons were performed between the control and treatment groups using Tukey's *post hoc* test for multiple comparison of one-way analysis of variance using GraphPad Prism 5.0 (GraphPad Software Inc., San Diego, CA, USA). *P*<0.05 was considered statistically significant.
